# *Saprinus planiusculus* (Motschulsky‚ 1849) (Coleoptera: Histeridae), a beetle species of forensic importance in Khuzetan Province, Iran

**DOI:** 10.1186/s41935-017-0004-z

**Published:** 2017-07-18

**Authors:** M. R. Fakoorziba, M. Assareh, D. Keshavarzi, A. Soltani, M. D. Moemenbellah-Fard, M. Zarenezhad

**Affiliations:** 1 Research Centre for Health Science, Institute of Health, Department of Medical Entomology and Vector Control, School of Health, Shiraz University of Medical Sciences, Shiraz, Iran; 2 Department of Medical Entomology and Vector Control, School of Public Health, Tehran University of Medical Sciences, Tehran, Iran; 3 Legal Medicine Research Centre, Legal Medicine Organization, Tehran, Iran

## Abstract

**Background:**

Medico legal forensic entomology is the science and study of cadaveric arthropods related to criminal investigations. The study of beetles is particularly important in forensic cases. This can be important in determining the time of death and also obtain qualitative information about the location of the crime. The aim of this study was to introduce the *Saprinus planiusculus* on a rat carrion as a beetle species of forensic importance in Khuzestan province.

**Methods:**

This study was carried out using a laboratory bred rat (Wistar rat) as a model for human decomposition. The rat was killed by contusion and placed in a location adjacent to the Karun River. Observations and collections of beetles were made daily during May to July 2015.

**Results:**

Decomposition time for rat carrion lasted 38 days and *S. planiusculus* was seen in the fresh to post decay stages of body decomposition and the largest number of this species caught in the decay stage.

**Conclusion:**

The species of beetle found in this case could be used in forensic investigations, particularly during the warm season in the future.

## Background

Medico legal forensic entomology is the science and study of cadaveric arthropods related to criminal investigations (Catts and Goff 1992). The study of beetles is particularly important in forensic cases. This can be helpful in determining the time since death or post-mortem interval (PMI) and also obtain qualitative information about the location of the crime (Matuszewski et al. 2008; Byrd and Castner 2009). Insects attracted to the chemical cues emitted by a dead body. They have a specific faunal succession to attack a decomposing body or exuded biological fluids‚ as carrion beetles are usually found in the late stages of body decomposition (Byrd and Castner 2009). Two major groups of insects are predictably attracted to remains and provide useful evidence in forensic investigation; the flies and the beetles (Catts and Goff 1992).

A variety of beetle species are necrophilous, or specifically attracted to carrion while not feeding on the carrion itself (Mashaly 2017; Rivers and Dahlem 2013).

The order Coleoptera comprises a number of forensically important families, viz. Staphylinidae, Nitidulidae, Scarabaeidae, Silphidae, Dermestidae, and Histeridae (Byrd and Castner 2009; Rivers and Dahlem 2013). Histeridae is a family of beetles commonly known as Clown beetles or *Hister* beetles (Catts and Goff 1992). Clown beetles are usually small, seldom getting beyond 10 mm in length. Both the larvae and adults are predacious and feed readily on juicy maggots and fly puparia (Byrd and Castner 2009). Histeridae are a large family with more than 3,502 identified species. They are frequent in tropical and subtropical climates. Their important diagnostic characters include geniculate and capitate antennae that are folded into a pronotal groove at repose (Bald 1935).

The legs are short and retracted, the fore tibia is fossorial, and the middle tibia frequently has long spines. The males have a hyaline membrane between the claws of fore tarsi. The abdomen has 5 visible sternites. Elytra do not cover the entire abdomen so that the apical two tergites are visible from above. Elytra are usually striate and punctuate (Geden and Axtell 1988).

The Histeridae family are predators that inhabit animal dung and carrion where they feed on other insects (Daria et al. 2011). Adults and immatures of Histeridae are found in association with decaying animal or vegetable matter, which suggested that they were principally scavengers. But it is becoming generally recognized that many species are predaceous on various insects (Ohara 2003).

Because the Histeridae family can be found on carrion, they have proven to be important in certain forensic investigations (Ozdemir and Osman 2009). The predacious Hister beetles will feed on the various insects on the body, primarily Diptera. To estimate a person’s time of death, forensic investigators must look at the insects on the body and determine the time of colonization (Kulshrestha and Satpathy 2001). If the Histeridae beetles are present, the investigator can assume that some of the other insects have been eaten by the Hister beetles.

## Methods

### Study site

**Fig. 1:**
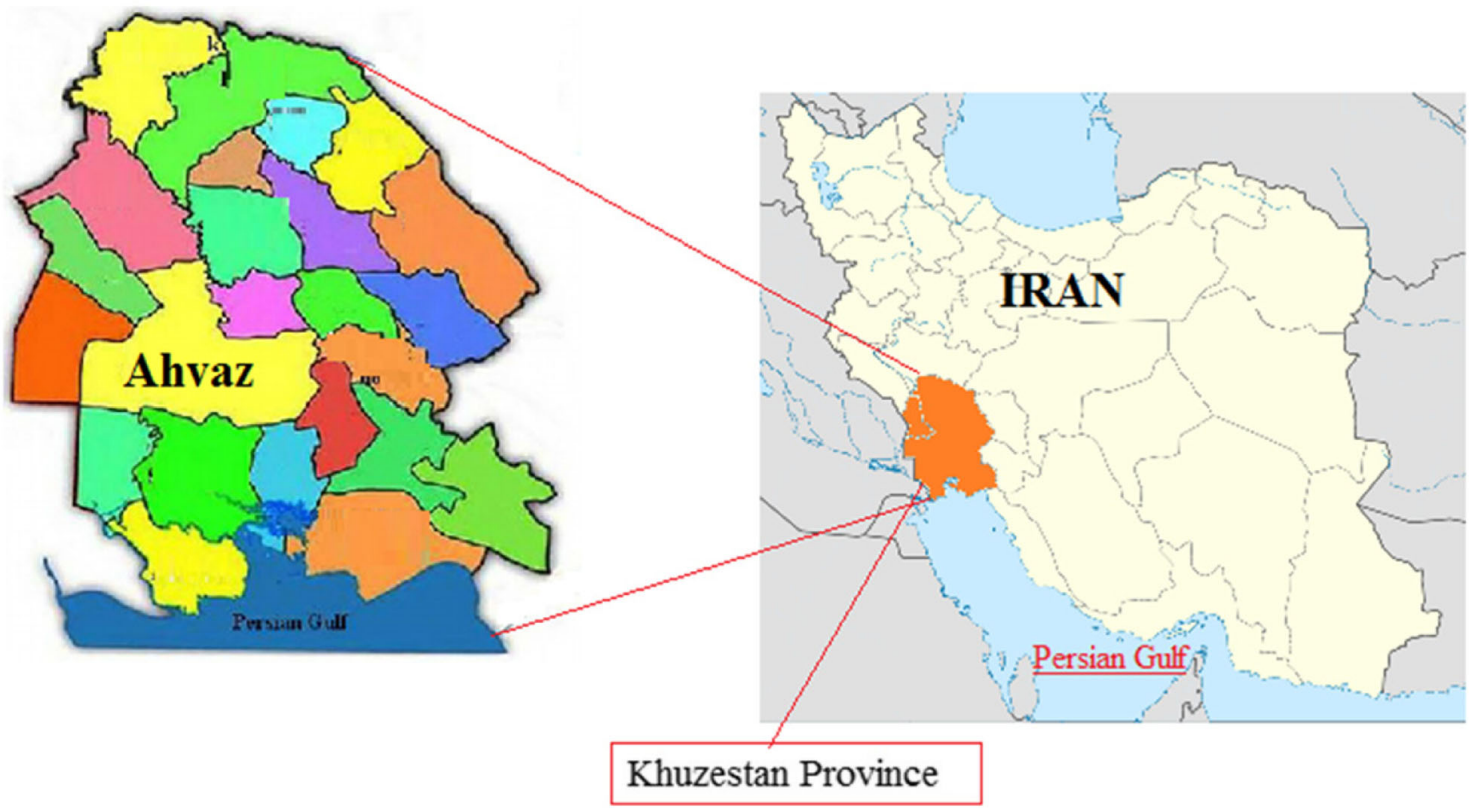
Map of Khuzestan province, south of Iran

**Fig. 2:**
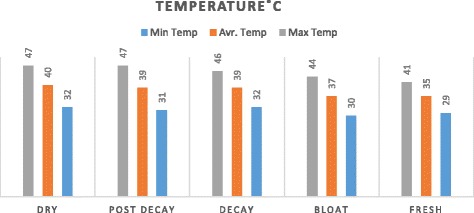
Changes in maximum and minimum ambient temperature between fresh and dry stages, Ahvaz City, May to July 2015

The study was carried out in a riverside location (Karun River) in Ahvaz city‚ Iran. Ahvaz is the capital of Khuzestan Province, located in the southern part of the country and bordering Iraq and the Persian Gulf (Fig. 1). The climate of Khuzestan is generally very hot and occasionally humid, particularly in the south, while winters are much more cold and dry. Summertime temperatures routinely exceed 48 °C and in the winter can drop below freezing, with occasional snowfall, all the way south to Ahvaz. The averages of minimum and maximum temperatures at the time of this study for each decomposition stage shown in Fig. 2.

### Study animal and insect collection

**Fig. 3:**
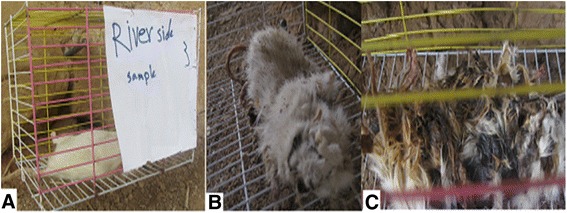
*Wistar rat* decomposition stages (**a**. Fresh stage, **b**. decay stage, **c**. remain)

This study was carried out using a laboratory bred rat (Wistar rat) weighing 352 g as a model for human decomposition (Fig. 3). The mouse was killed by contusion and placed in a location adjacent to the Karun River. Observations and collections of beetles were made once at night and once a day during May to July 2015. For identification, the body parts were carefully removed and dissected, then placed into 10% KOH solution. They were subsequently warmed in the liquid for about 45 min at 80 °C, rinsed with 80% ethanol, and dehydrated in 99% ethanol; some of them were stained in citric acid containing acid fuchsine and warmed in the liquid for 15 min at 60 °C before being rinsed. A valid taxonomic key was used for the identification of beetle species (Halstead 1963).

## Results

In this period of time‚ two guilds of carrion-dwelling insects; adult beetles in the family of Histerida (Coleoptera) and fly maggots of the Calliphoridae family were collected from the soil surface near the carrion. *Saprinus planiusculus* adults were collected only during the night. Decomposition time for the mouse lasted 38 days. *S. planiusculus* was seen in the fresh to post decay stages of body decomposition and the largest number of this species caught in the decay stage.

### *Saprinus planiusculus* (Motschulsky‚ 1849)

#### Distribution

Nearly throughout the Palaearctic Region (Daria et al. 2011).

#### Morphological descriptions

**Fig. 4:**
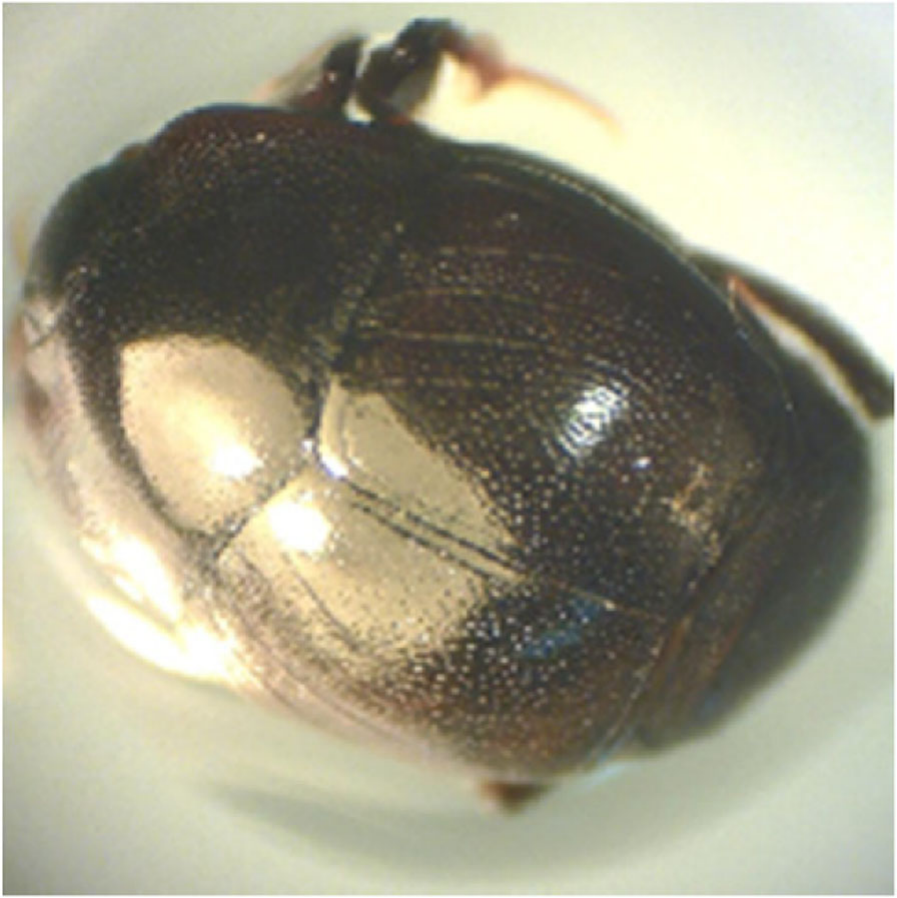
The habitus of *Saprinus planiusculus* (Histeridae family)

**Fig. 5:**
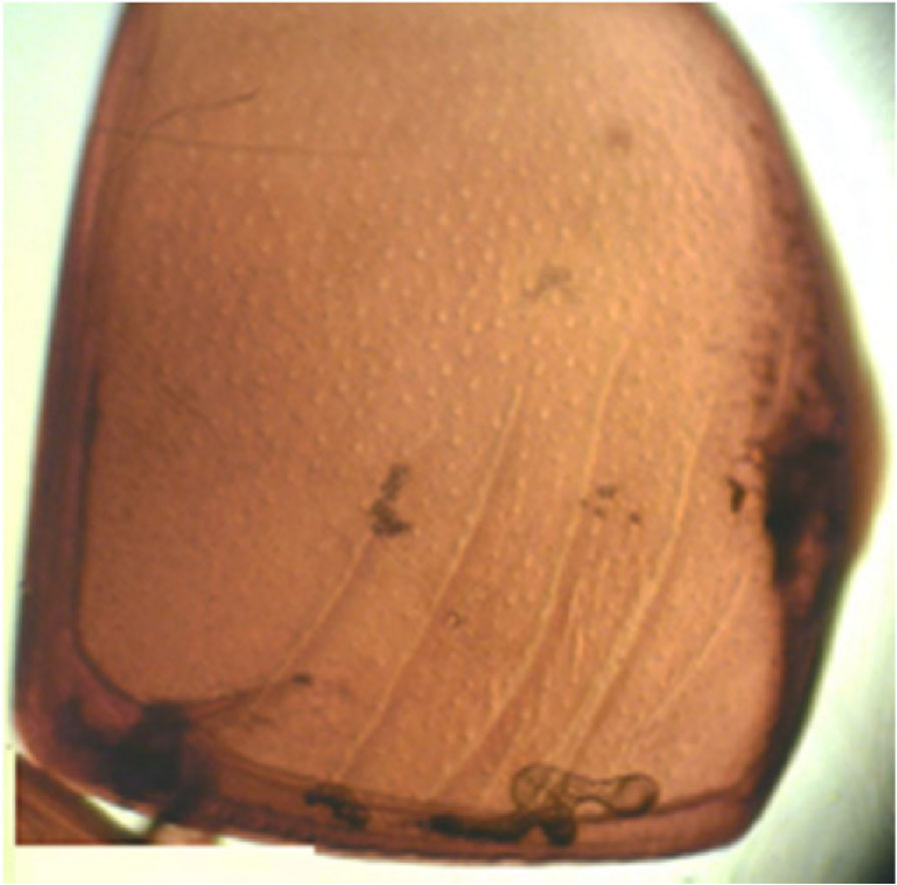
Elytra of *Saprinus planiusculus* with moderate punctures

Body length‚ male: 3.95-5.66 mm, female: 4.09-6.33 mm. Top of pronotum without punctures or only with very small punctures, color black‚ frontal stria of head weakly carinate and complete (Fig. 4). The supraorbital stria well impressed and complete. Disk of front densely covered with moderate punctures, which become sparser on the basal half; interspaces among these punctures usually smooth, occasionally clothed with fine punctures. Epistoma medio-apically more densely punctate than the disk‚ labrum deeply depressed medially‚ epipleura of elytra sparsely with moderate punctures, which become denser on the apical third (Fig. 5). Marginal epipleural stria complete and finely impressed. Marginal elytral stria lightly carinate and complete, its apical end extending along the posterior margin of elytra to medio-apical angles of elytra and then bending basally and running for a short distance. External subhumeral stria confined basally. Anterior margin of mesosternum broadly and feebly emarginate medially. Marginal stria complete, and strongly carinate. Protibia with 13 spines on outer margin, the apical two and basal three small. *Saprinus planiusculus* resembles *Saprinus niponicus*, with which it is to a great extent sympartic; however, it can be distinguished from the latter by the shortened third dorsal elytral stria and the shape of the 8th sternum of the male genitalia (Ohara 1994, 2003; Yélamos 2002).

## Discussion

Since insect live on decomposing body tissues both as a food source and/or habitat they could be manipulated to estimate the postmortem interval in line with the data on species of insect, their growth rates and fluctuating environmental temperatures (Byrd and Castner 2009; Ohara 1994; Keshavarzi et al. 2016).

Predatory insects can intercept intraspecific pheromones to discover necrophagous species on the same odors of decomposition used by saprophagous insects to find the carrion that is home to thousands of individuals that may well become their next meal or host (Rivers and Dahlem 2013).

*S. planiusculus* has been recorded from carrions in Poland and Turkey (Matuszewski et al. 2008; Daria et al. 2011; Rozner 2010). A study in Central Europe showed that this species mainly bred in open habitats and rarely bred in forests (Matuszewski et al. 2013). In Central Europe this species bred in cadavers from spring to summer (Matuszewski et al. 2013; Mądra et al. 2015). This result is similar with our finding. Some species of Calliphoridae family associated with carcasses were found in Iran (Keshavarzi et al. 2015a, 2015b, 2015c). Three families of beetles (Histeidae, Dermestidae and Staphylinidae) previously were reported from southern Iran (Fereidooni et al. 2015).

The study on the life history patterns of insects with forensic importance is essential and plays a important role in forensic entomology field for PMI determination (Keshavarzi et al. 2015c). Hister beetles have a complete metamorphosis and life cycle consists of an egg stage, three to five larval stages, and a pupal stage before becoming an adult. The number of instars in the larval stage is depends on the species and environmental conditions (Catts and Goff 1992; Rivers and Dahlem 2013). They are predacious and feed freely on insect larvae and fly puparia (Byrd and Castner 2009). The average time of development for this family from egg to imago at 30°c is about 20 days (Peter and Kovarik 2001). Development from egg to adult averaged 26.3 days at 27 °C in *Hister abbreviatus* (Summerlin et al. 1984). There is no detailed information about S. *planiusculus* lifecycle. Based on our observations, this insect is active at night and during the day hiding in the soil and feed on the insects that are present on the body. Therefore, in forensic entomology studies, behavior and food habits of S. *planiusculus* should be considered.

## Conclusion

This beetle is active in fresh to post decay stages of body decomposition and can be helpful for the development of forensic entomology field in Iran. Future research will be needed on the biology and ecology of S. *planiusculus*, in order to determine the exact length of each life cycle stages, which will be of great value to the further improvement of the PMI estimation and other forensic implications.

## References

[CR1] Bald WV (1935). The Bionomics of Entomophagous Coleoptera.

[CR2] Byrd JH, Castner JL (2009). Forensic Entomology: The Utility of Arthropods in Legal Investigations.

[CR3] Catts EP, Goff ML (1992). Forensic entomology in criminal investigations. Annu Rev Entomol.

[CR4] Daria B, Matuszewski S, Konwerski S (2011). Insect succession on carrion: seasonality, habitat preference and residency of histerid beetles (Coleoptera: Histeridae) visiting pig carrion exposed in various forests (Western Poland). Pol J Ecol.

[CR5] Fereidooni M (2015). Preliminary Data on Life Cycle of Creophilus Maxillosus Linnaeus (Coleoptera: Staphylinidae) and New Report of this Species on a Human Corpse, South of Iran. Int J Forensic Sci Pathol.

[CR6] Geden CJ, Axtell R (1988). Predation by *Carcinops pumilio* (Coleoptera: Histeridae) and *Macrocheles muscaedomesticae* (Acarina: Macrochelidae) on the housefly (Diptera: Muscidae): Functional response, effects of temperature and availability of alternative prey. Environ Entomol.

[CR7] Halstead D (1963). Handbooks for the Identification of British Insects. R Entomol Soc.

[CR8] Keshavarzi D, Fereidooni M, Assareh M, Nasiri Z (2015). A checklist of forensic important flies (Insecta: Diptera) associated with indoor rat carrion in Iran. J Entomol Zool Stud.

[CR9] Keshavarzi D, Moemenbellah-Fard MD, Fereidooni M, Montazeri M (2015). First Report of Dermestes *Frischii* Kugelann (Coleoptera: Dermestidae) on a Human Corpse, South of Iran. Int J Forensic Sci Pathol.

[CR10] Keshavarzi D, Moemenbellah-Fard MD, Fereidooni M, Zarenezhad M, Fakoorziba M (2015). R. New record of sap beetle, Nitidula flavomaculata Rossi (Coleoptera: Nitidulidae) on an outdoor mummified human corpse, South of Iran. J Entomol Zool Stud.

[CR11] Keshavarzi D, Moemenbellah-Fard MD, Zarenezhad M, Gholamzadeh S (2016). First Forensic Record of Blowfly, Calliphora vicina, Larvae on an Indoor Human Corpse in Winter, South of Iran. Int J Forensic Sci Pathol.

[CR12] Kulshrestha P, Satpathy DK (2001). Use of beetles in forensic entomology. Forensic Sci Int.

[CR13] Mądra A, Frątczak K, Grzywacz A, Matuszewski S (2015). Long-term study of pig carrion entomofauna. Forensic Sci Int.

[CR14] Mashaly AM (2017). Carrion beetles succession in three different habitats in Riyadh, Saudi Arabia. Saudi J Biol Sci.

[CR15] Matuszewski S, Bajerlein D, Konwerski S, Szpila K (2008). An initial study of insect succession and carrion decomposition in various forest habitats of Central Europe. Forensic Sci Int.

[CR16] Matuszewski S, Szafałowicz M, Jarmusz M (2013). Insects colonising carcasses in open and forest habitats of Central Europe: search for indicators of corpse relocation. Forensic Sci Int.

[CR17] Ohara M (1994). A Revision of the superfamily Histeroidea of Japan (Coleoptera). Ins. Matsum.n. s.

[CR18] Ohara M (2003). Notes on Taiwan Species of the genus Sapprinus (Coleoptera: Histerida). Insecta matsumurana. Ser Entomol.

[CR19] Ozdemir S, Osman S (2009). Determination of Coleoptera fauna on carcasses in Ankara province, Turkey. Forensic Sci Int.

[CR20] Peter W, Kovarik MS, Arnett H, Michael J, Thomas C (2001). Histeridae, in Ross. American Beetles.

[CR21] Rivers DB, Dahlem GA (2013). The science of forensic entomology.

[CR22] Rozner I (2010). Additional data to the hister beetle fauna of Turkey (Coleoptera: Histeridae). Nat Somogyiensis.

[CR23] Summerlin JW, Bay DE, Stafford KC, Hunter JS (1984). Laboratory observations on the life cycle and habits of Hister abbreviatus (Coleoptera: Histeridae). Ann Entomol Soc Am.

[CR24] Yélamos T (2002). Coleoptera, Histeridae.

